# Supplementary material for: Does Supramolecular Gelation Require an External Trigger?
Source: Gels. 2022 Dec 10;8(12):813. doi: 10.3390/gels8120813 (PMC9778329; doi:10.3390/gels8120813)
Supplement: Supplementary file 1 [file gels-08-00813-s001.zip › gels-2014545-supplementary.pdf]

Supplementary Information for:

Does Supramolecular Gelation Require an External Trigger?

Ruben Van Lommel [1,2], Julie Van Hooste [1], Johannes Vandaele [3], Gert Steurs [1], Tom Van der Donck [4], Frank De Proft [2], Susana Rocha [3], Dimitrios Sakellariou [5], Mercedes Alonso [2,*] and Wim M. De Borggraeve [1,*]

[1] Molecular Design and Synthesis, Department of Chemistry, KU Leuven, Celestijnenlaan 200F, box 2404, 3001 Leuven, Belgium
[2] Eenheid Algemene Chemie (ALGC), Department of Chemistry, Vrije Universiteit Brussel (VUB),
Pleinlaan 2, 1050 Brussels, Belgium
[3] Molecular Imaging and Photonics, Department of Chemistry, KU Leuven, Celestijnenlaan 200F, box 2404, 3001 Leuven, Belgium
[4] Department of Materials Engineering, KU Leuven, Kasteelpark Arenberg 44, 3001 Leuven, Belgium
[5] Center for Membrane Separations, Adsorption, Catalysis and Spectroscopy for Sustainable Solutions (cMACS), Department of Microbial and Molecular Systems (M2S), KU Leuven, Celestijnenlaan 200F,
box 2454, 3001 Leuven, Belgium
* Correspondence: mercedes.alonso.giner@vub.be (M.A.); wim.deborggraeve@kuleuven.be (W.M.D.B.)

Table of Contents

S1. Scanning electron microscopy

S1.1 General details S3

S1.2 Multi-stimuli responsiveness S3

S1.3 Images S4

S2. Single particle tracking experiments

S2.1 General details S7

S2.2 Results S7

S2.3 Movies S8

S3. Molecular Dynamics simulations

S3.1 General details S8

S3.2 Analysis S9

S4. NMR Experiments

S4.1 General details S10

S4.2 ^{13}C solution-state spectrum S11

S4.3 ^{1}H-^{13}C HSQC spectrum S12

S4.4 ^{1}H-^{13}C HMBC spectrum S12

S4.5 ^{1}H-^{15}N HSQC spectrum S13

S4.6 ^{1}H-^{13}C HSQC at 60°C S13

S4.7 VT-NMR experiments S14

S4.8 NOESY spectrum at 60 °C S15

S4.9 VC-NMR experiments S15

S4.10 QNMR through ERETIC2 *S16*
S4.11 NMR experiments below MGC *S19*
S5. References and notes *S19*

S1. Scanning electron microscopy

S1.1. General details

Sample preparation to obtain the scanning electron microscopy (SEM) images of the xerogel started by preparing the corresponding **p2*o*1** hydrogel using a variety of gelation triggers which are explained in detail below. Next, water is removed to obtain the xerogel by freezing the sample using liquid nitrogen and subsequent freeze-drying. Under these cryogenic conditions, possible drying effects are minimized. Using a toothpick, a small amount of the bulk xerogel is placed on a conductive carbon adhesive disc, which is coated with Pt (5 nm) using a Quorum Q150T S coater system. Backscattered electron images are obtained using a FEI Nova NanoSEM 450 FEG SEM with a concentric backscatter detector (CBS) operating at a landing energy voltage of 5 kV (using Beam Deceleration, 4 kV). Scattered electron images were taken using the same system and acceleration voltage, but with a through-the-lens-detector (TLD) in immersion mode or Everhart-Thornley detector (ED) in field free mode.

S1.2. Multi-stimuli responsiveness

Besides heat-triggered and spontaneous gelation which are described in the main article, a variety of other triggers can be used to obtain the **p2*o*1** hydrogel such as: pH and sonication. A schematic overview of possible gelation procedures for **p2*o*1** is provided in Figure S1. The triggers presented here achieve a re-organization of the gelator molecules towards a supramolecular gel network by disrupting the urea-urea hydrogen bonds that were already present in the solid non-gel phase. This can be achieved by providing sufficient energy for the hydrogen bonds to break (heat trigger, sonication) or by creating strong electrostatic repulsive interactions between the gelator molecules (pH). Most gelation procedures proceed through an unsaturated solution phase where all intermolecular connections between the gelators are disconnected. However, this does not necessarily have to be the case, *e.g.* when sonication is used as a trigger no clear solution is reached during the procedure.
To compare the supramolecular hydrogels of **p2*o*1** that were obtained from different triggers, back-scattered electron (BSE) images were taken from their corresponding xerogels (see Section 1.3 in Supplementary Information). When pH or sonication is used as trigger, similar architectures (nanospheres and fibers) are observed compared to when a heat-trigger is used. This suggests a comparable stepwise hydrogelation mechanism to occur. Albeit some structural differences between the xerogels are observable. Indeed, for example the nanospheres observed for the xerogel obtained through a pH-trigger are more irregular which might be attributed to the presence of sodium and chloride ions that change the electrostatic properties of the medium. Furthermore, the sheet-like structures for the xerogel obtained through sonication are less extensive due to a smaller entanglement density.

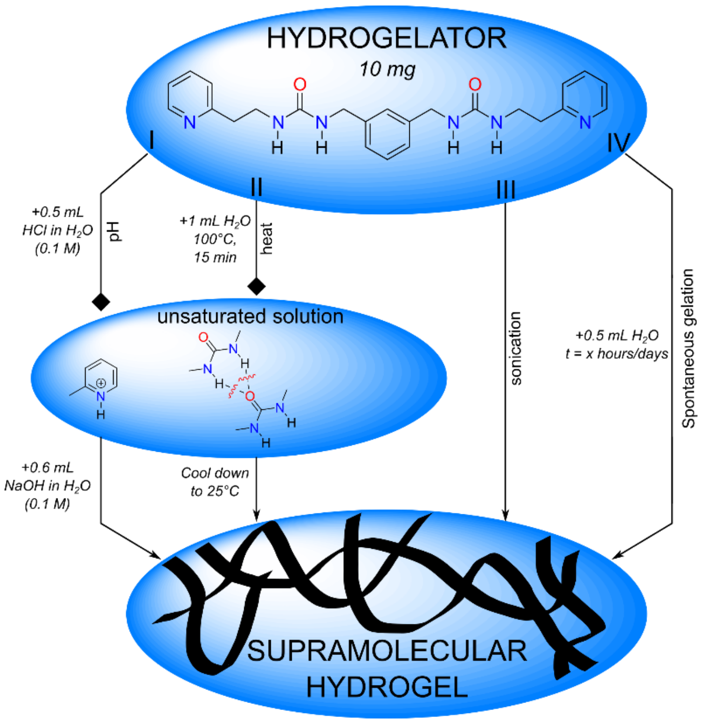

Figure S1. *Schematic overview of possible gelation procedures to obtain a multi-responsive hydrogel.*

S1.3. Images

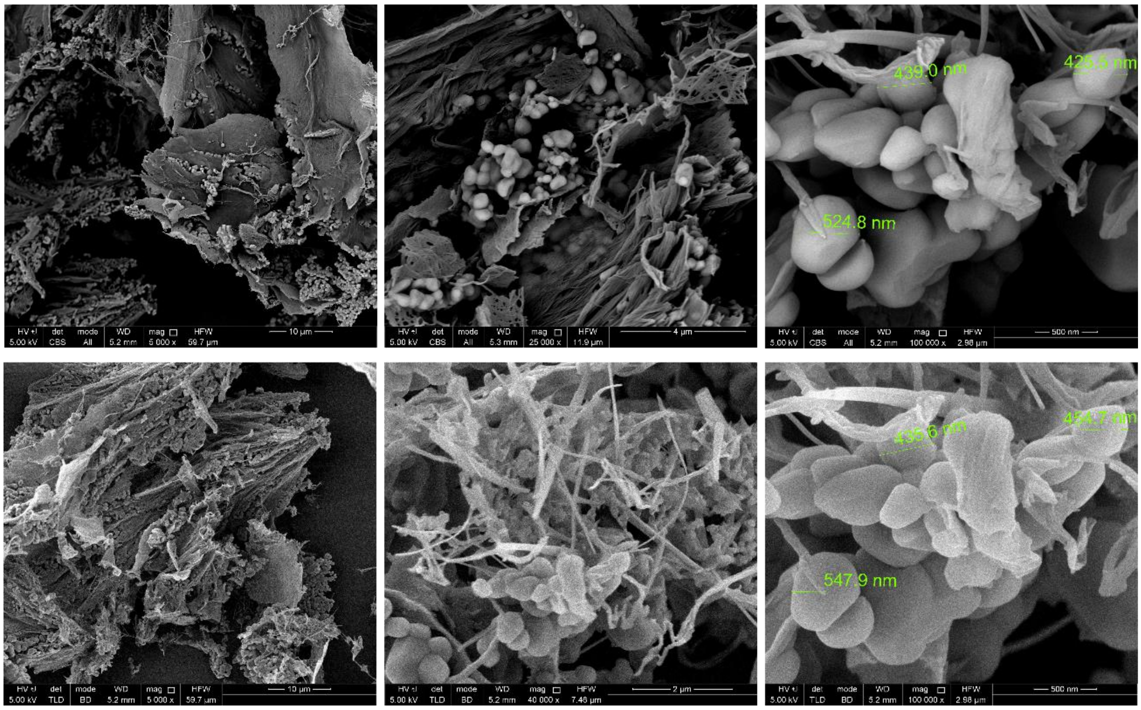

Figure S2. *Back-scattered electron images (top) and scattered electron images (bottom) of the* ***p2o1*** *xerogel prepared using a pH-trigger (method I) at different magnifications. The scale-bar is provided at the bottom-right of each image.*

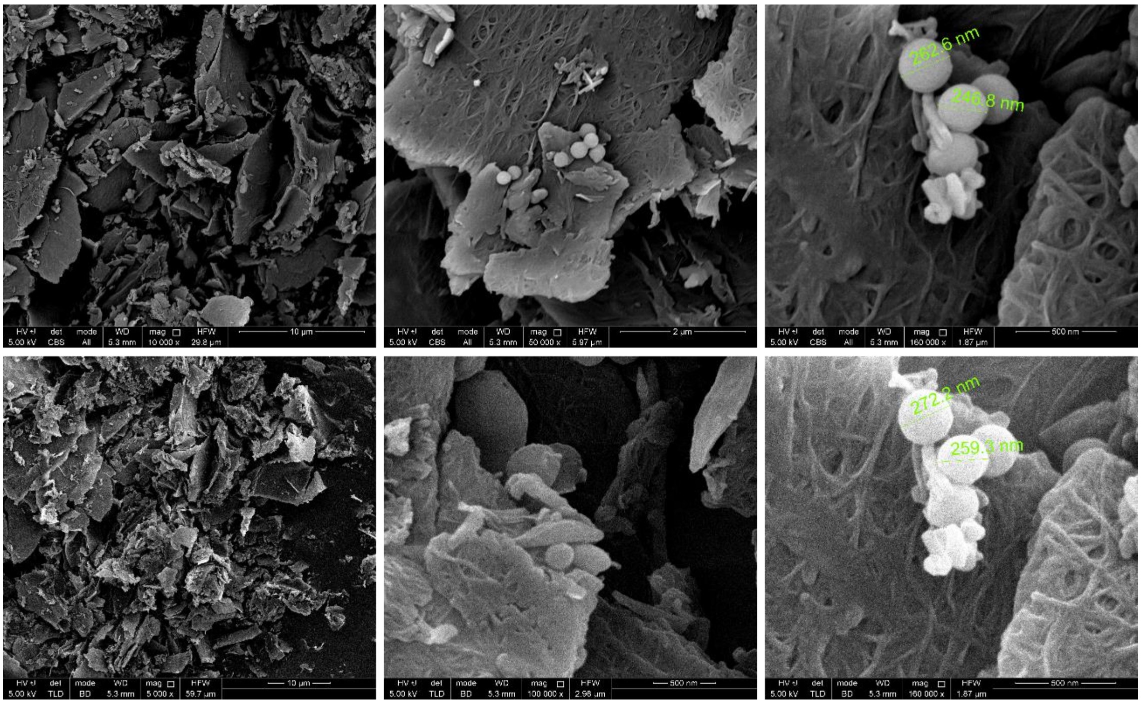

Figure S3. *Back-scattered electron images (top) and scattered electron images (bottom) of the* ***p2o1*** *xerogel prepared using a heat-trigger (method II) at different magnifications. The scale-bar is provided at the bottom-right of each image.*

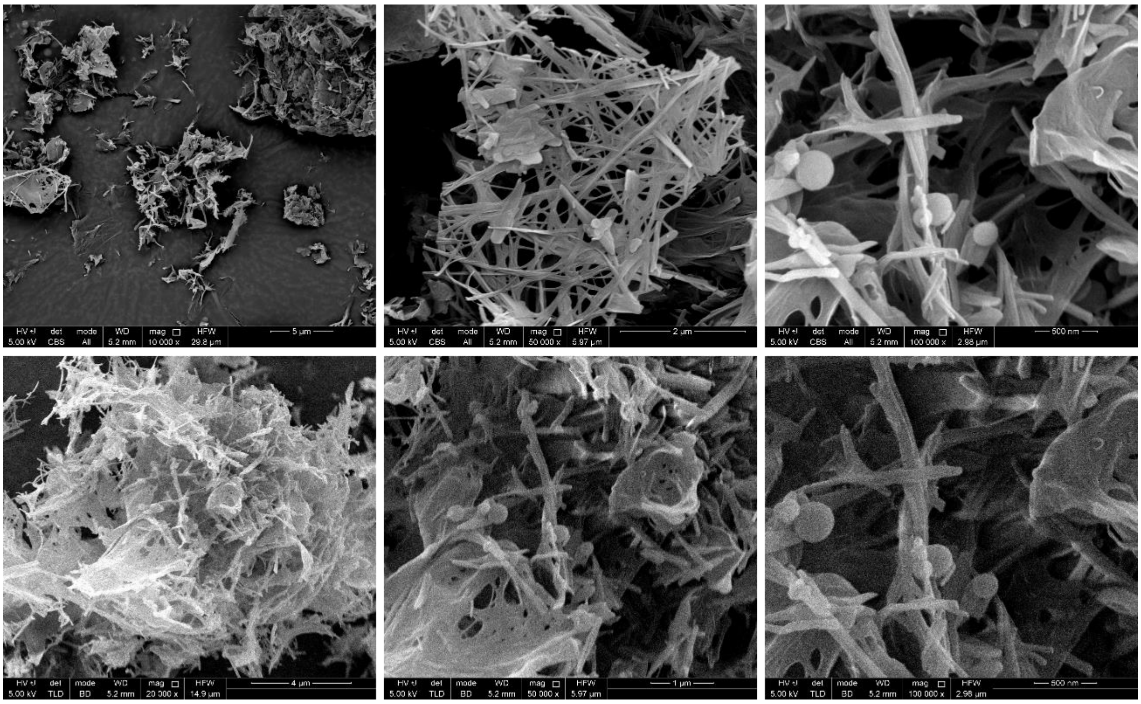

Figure S4. *Back-scattered electron images (top) and scattered electron images (bottom) of the* ***p2o1*** *xerogel prepared using a sonication trigger (method III) at different magnifications. The scale-bar is provided at the bottom-right of each image.*

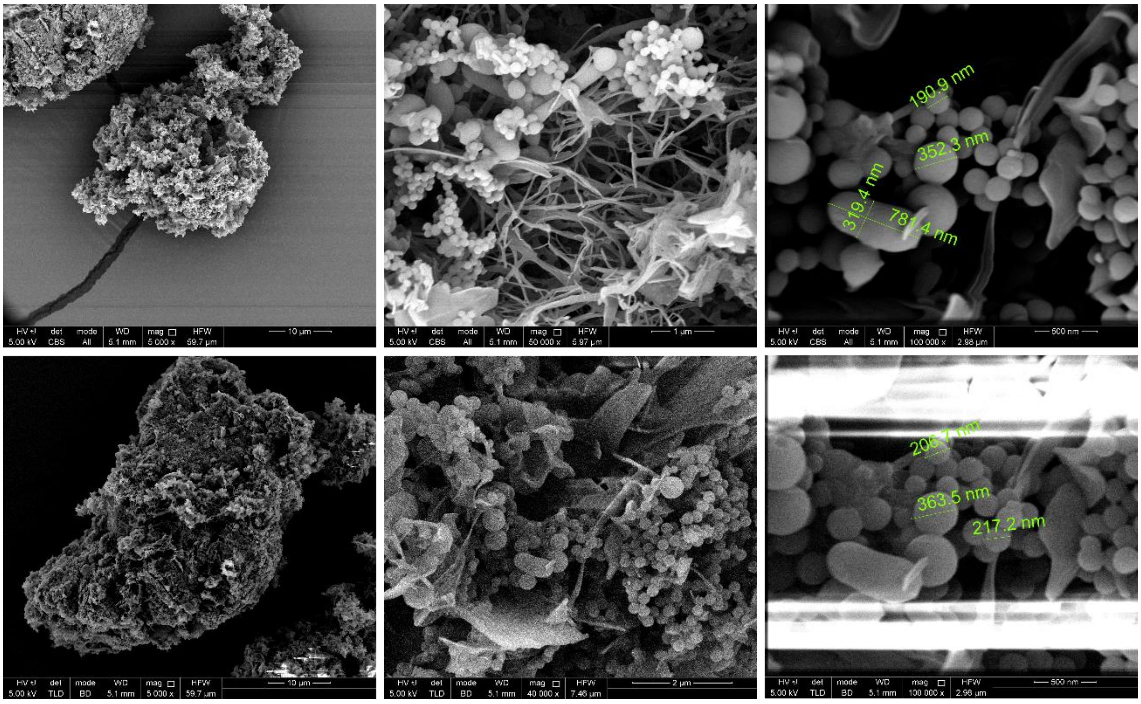

Figure S5. *Back-scattered electron images (top) and scattered electron images (bottom) of the* ***p2o1*** *xerogel prepared without trigger after 1 hour (method IV, t = 1 hour) at different magnifications. The scale-bar is provided at the bottom-right of each image.*

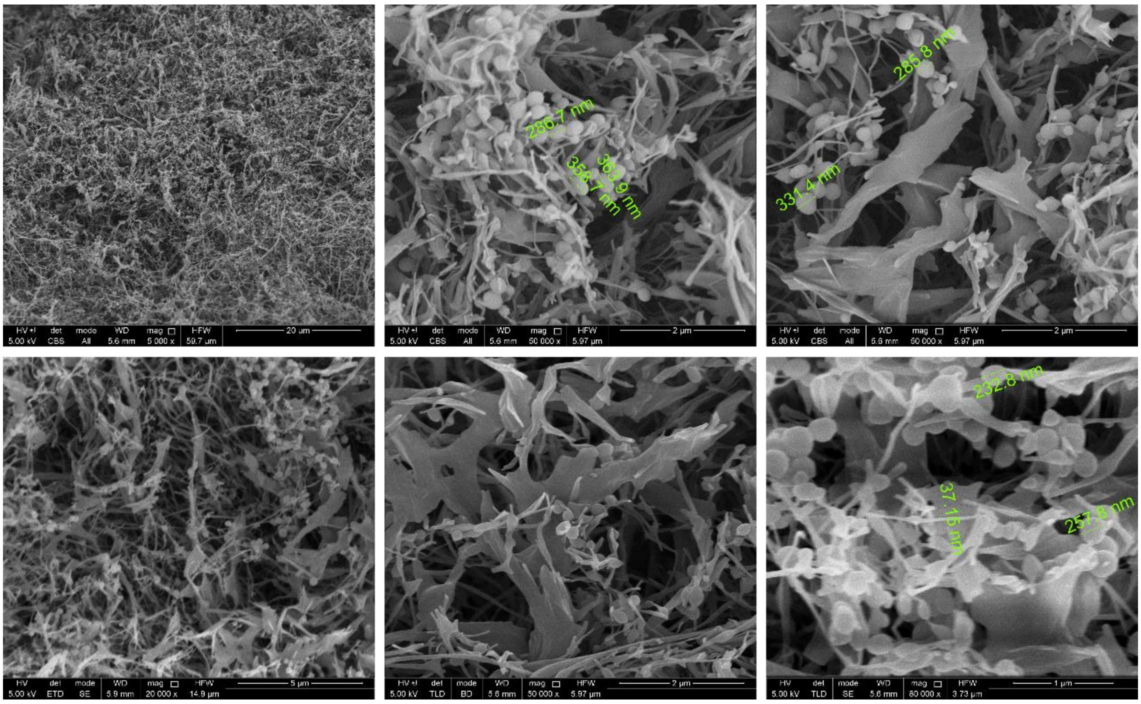

Figure S6. *Back-scattered electron images (top) and scattered electron images (bottom) of the* ***p2o1*** *xerogel prepared without trigger after 14 days (method IV, t = 14 days) at different magnifications. The scale-bar is provided at the bottom-right of each image.*

S2. Single particle tracking experiments

S2.1. General details

The custom designed setup used for these experiments is fully described in other work [1]. All the Matlab-scripts used to analyze the data are available online for free on Github (https://github.com/CamachoDejay) and can be downloaded and directly imported as a functional script into Matlab. Plotting of data was done using Microsoft Excel. For the experiments, 10 mg/mL and 5 mg/mL solutions of the gelator in de-ionized water were used. All solutions were freshly prepared right before imaging. Two types of gelation were studied: spontaneous and heat-triggered gelation.

For the spontaneous gelation experiments, 7 movies were acquired per time point, each time at random places in the sample and at least 30 µm above the glass bottom. The first time point is 'Hour 0', measured directly after the sample was prepared. For each time point, all the data extracted from 7 individual movies were averaged resulting into 1 value, depicted in the plot (Figure S7 and S8) together with their standard deviation. This procedure was repeated until time point 'Hour 6' was reached. To assure reproducibility, each experiment was conducted in triplicate. The final plot shown in the main article is the result of an average of 3 individual experiments.

For the heat triggered experiments, 2 movies were acquired at each time point over a total timespan of 25 minutes. Before measuring, the sample was brought to 100 °C and heated for 5 minutes. Directly afterwards the first time point, '0 minutes', was measured. At each time point, 2 movies were acquired at random places and at least 30 µm above the glass bottom. Per time point the data extracted from two corresponding movies were averaged and plotted together with corresponding standard deviation (Figure S7 and S8). The final plot shown in the main article is the result of an average of 3 individual experiments.

S2.2. Results

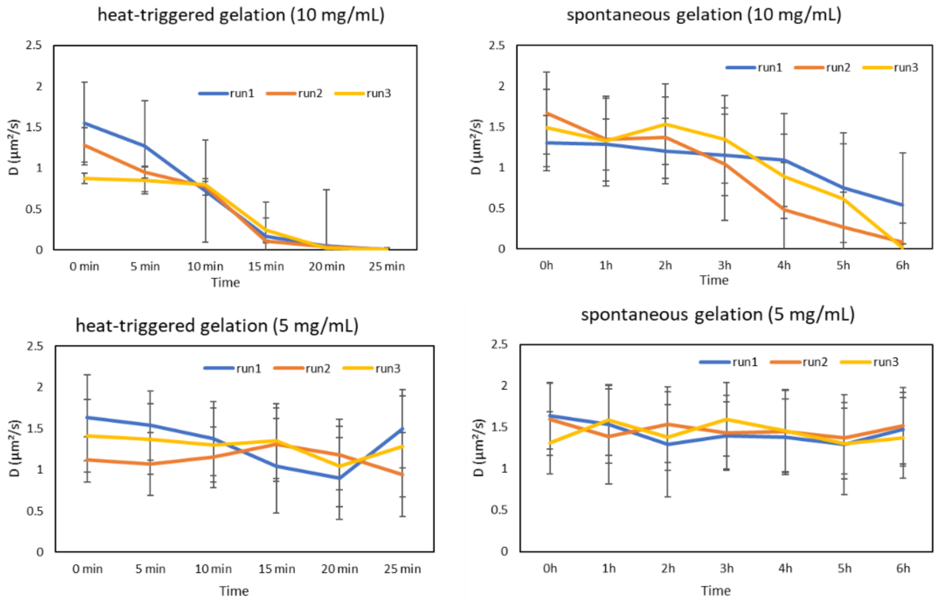

Figure S7. *Plots show the mean diffusion coefficients of the beads obtained from different measurements. In top left and top right panels the gelation is studied for a gelator solution of 10 mg/mL in water for both heat-triggered and spontaneous gelation, respectively. In bottom left and right panel the same gelation studies were performed but for a gelator concentration of 5 mg/mL.*

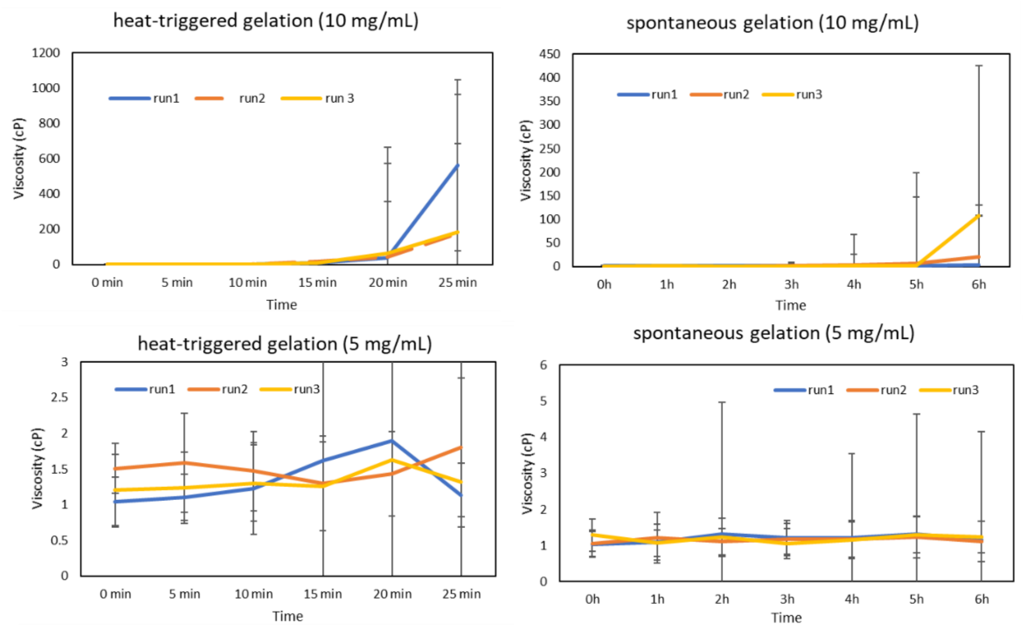

Figure S8. *Plots show the mean viscosity values obtained from different measurements. In top left and top right panels the gelation is studied for a gelator solution of 10 mg/mL in water for both heat-triggered and spontaneous gelation, respectively. In bottom left and right panel the same gelation studies were performed but for a gelator concentration of 5 mg/mL.*

S2.3. Movies

As an example, we included a short movie from an experiment where we monitored spontaneous gelation of a 10 mg/mL gelator solution. The movie shows 500 acquired frames at a speed of 30 frames per second. Two sets of movies are displaying each 4 channels on camera 1 (plane 1 to 4). The top set represents the initial state of the experiment, while the bottom set represents the 6-hour mark after spontaneous gelation takes place. The exposure time is 0.020 seconds for all the acquired movies. From the top set, we can see a random diffusion of the fluorescent beads. After 6 hours, we can clearly see that the fluorescent beads are now in a more stationary phase, distributed in 3 dimensions throughout the sample.

S3. Molecular Dynamics simulations

S3.1. General details

Molecular dynamics (MD) simulations in this study were performed in triplicate using the Gromacs software (version 2018.3) [2]. Three simulation boxes were created by placing 100 fully extended **p2*o*1** gelator molecules in a periodic cubic box with a box edge of 15.74556 nm. The gelator molecules were fully dispersed in the simulation box, *i.e.* in the initial topology no intermolecular noncovalent interactions were present between the **p2*o*1** molecules. This was accomplished by setting the initial minimum distance between two different gelators at 3.0 Å using the packmol code [3]. Subsequently, the simulation box was solvated with water molecules which were described by the TIP3P-CHARMM water model, reaching a **p2*o*1** concentration of 1.0% w/V, similar to the experimental concentration used in this work [4]. Trajectories were gathered using the CHARMM27 force field, with the parameters of the **p2*o*1** gelator obtained from the Swissparam service [5,6]. In our previous work, we validated the accuracy of the CHARMM27 force field together with the aforementioned parametrization, in describing the noncovalent interactions of small organic urea-based gelators [7].

Prior to production runs, each simulation underwent an energy minimization step and temperature and pressure equilibration step to eliminate steric clashes and ensure adequate equilibration of the NPT-

ensemble. The equilibration step consisted out of two stages. In the first stage, temperature is set at 303 K and controlled by the V-rescale thermostat with the time constant set at 0.1 ps, while the pressure is set at 1.0 bar and controlled by the Berendsen barostat with the time constant set at 2.0 ps [8]. The first equilibration ran for 1 ns with a timestep of 1 fs. The second equilibration stage differs from the first as the pressure is now set at 1.0 bar, but controlled by the Parrinello-Rahman barostat with a time constant of 2.0 ps [9]. Latter stage ran for 1 ns with a timestep of 2 fs. After the second equilibration stage, a production trajectory of 150 ns was obtained in the NPT-ensemble with a timestep of 2 fs. Temperature and pressure control were established similar to the second equilibration stage. Important properties, averaged over the 150 ns production run, can be found in Table S1.

Table S1. *Average temperature (in K), pressure (in bar) and density (in kg/m³) of the three 150 ns molecular dynamics simulation production runs.*

**Entry**	**Temperature (K)**	**Pressure (bar)**	**Density (kg/m³)**
**1**	302.9915	0.9976	985.6965
**2**	302.9902	0.9648	985.7044
**3**	302.9937	0.9614	985.6892

S3.2. Analysis

In previous work, we introduced 4 descriptors that are obtained through all-atom molecular dynamics simulations and which provide valuable information concerning the aggregation process of supramolecular gelators [10]. These descriptors were also applied in this study to analyze in more detail the molecular dynamics simulations. Below, the equations are provided to compute these descriptors, however for a detailed explanation on how to compute these values, we refer to the supporting information of our previous work [10].

$$(1) \qquad r_{SASA} = \frac{SASA}{SASA_{max}}$$

In equation 1, the *rSASA* descriptor is computed by taking the ratio of the solvent accessible surface area (*SASA*) over the maximum solvent accessible surface area ($SASA_{max}$). For 100 **p2*o*1** molecules, the latter value is 792 nm².

$$(2) \qquad rH = \frac{R}{R_{max}}$$

In equation 2, the relative end-to-end distance *rH* is obtained by taking the ratio of the distance between the atoms of a single gelator molecule which would be furthest apart from each other in an extended conformation (*R*), with the distance these atoms have in the fully extended conformation (R_{max}).

$$(3) \qquad F = \frac{R_g}{R_h{}'}$$

In equation 3, the shape factor *F* is obtained by taking the ratio of the radius of gyration of the gelator molecules in the simulation (R_g) over a pseudo hydrodynamic radius ($R_h{}'$). The latter value is equal to 3.155 nm for 100 **p2*o*1** molecules.

As this study focused on the evolution of the descriptors, rather than a single value that can be compared across different gelator-solvent systems, the usual HB% descriptor was replaced by a hydrogen bond count

across all gelators in the system. This hydrogen bond count can be directly obtained from the trajectory file (.xtc) in Gromacs using the following command line:

```
gmx_mpi hbond -f filename.xtc -s filename.tpr -n index.ndx  -r 0.3 -num hbond.xvg
```

S4. NMR experiments

S4.1. General details

Samples were prepared by heating a screw capped vial containing the gelator **p2*o*1** in a H_2O:D_2O mixture (varying ratio depending on the experiment) to 120 °C using a copper heating block. In general, the concentration of the gelator in H_2O:D_2O was set to 1.0% w/V for each experiment, unless specifically stated otherwise. The presence of D_2O is required for field-frequency locking. After 5 minutes of heating, when the gelator was fully dissolved, the warm solution was quickly added into a 5 mm NMR tube through a syringe. The gel was obtained after 30 minutes of cooling to room temperature. H_2O of ultrapure Milli-Q grade was used in all experiments, while D_2O was purchased from Sigma-Aldrich®. Furthermore, all NMR spectra discussed below were recorded on a Bruker Avance II+ 600 spectrometer operating at 14.1 Tesla with a 5 mm TXI H-C/N-D probe (for all ^{1}H-detected experiments) or a 5 mm BBO (^{31}P-^{103}Ag)-^{1}H/D probe (for ^{13}C-detected experiments), unless specifically stated otherwise. Samples for solution-state NMR experiments were prepared as described above with a H_2O:D_2O ratio of 9:1, 0:10 and 9:1 respectively for ^{1}H, ^{13}C and ^{15}N detections. For ^{1}H-NMR spectra, water suppression was accomplished using various water suppression blocks (details in captions) [11].

S4.2. ^{13}C solution-state spectrum

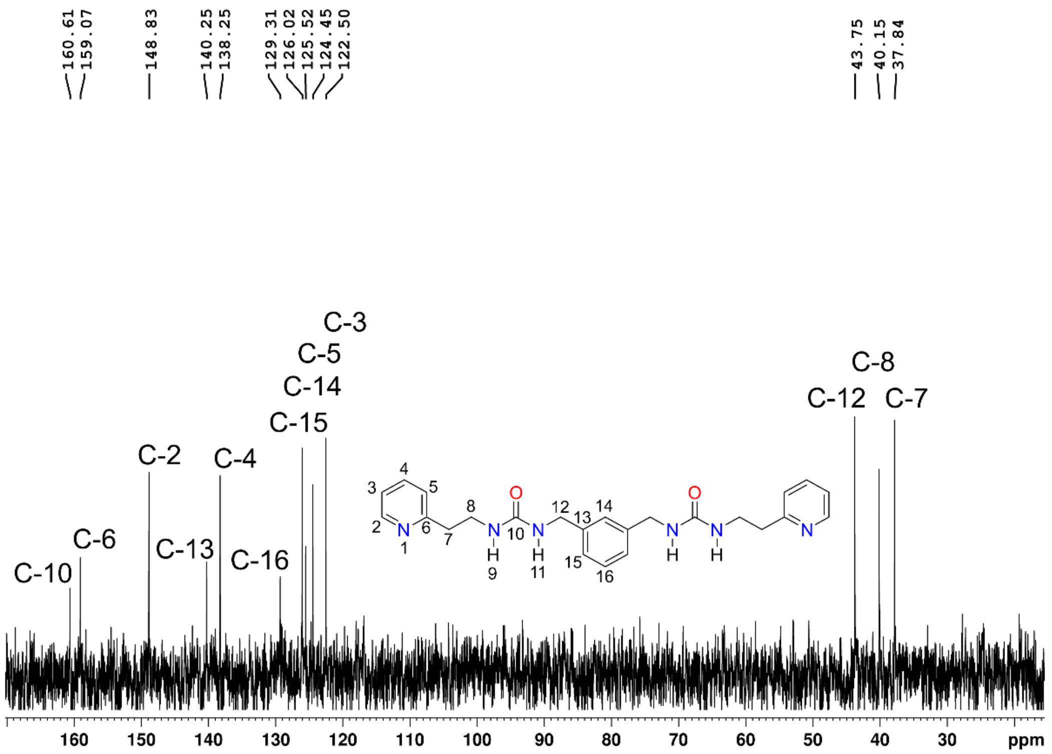

Figure S9. *^{13}C solution-state NMR spectrum of the **p2o1** hydrogel in pure D_2O at a concentration of 1% w/V. During acquisition, the number of scans was equal to 467, while a repetition delay of 2 seconds was used.*

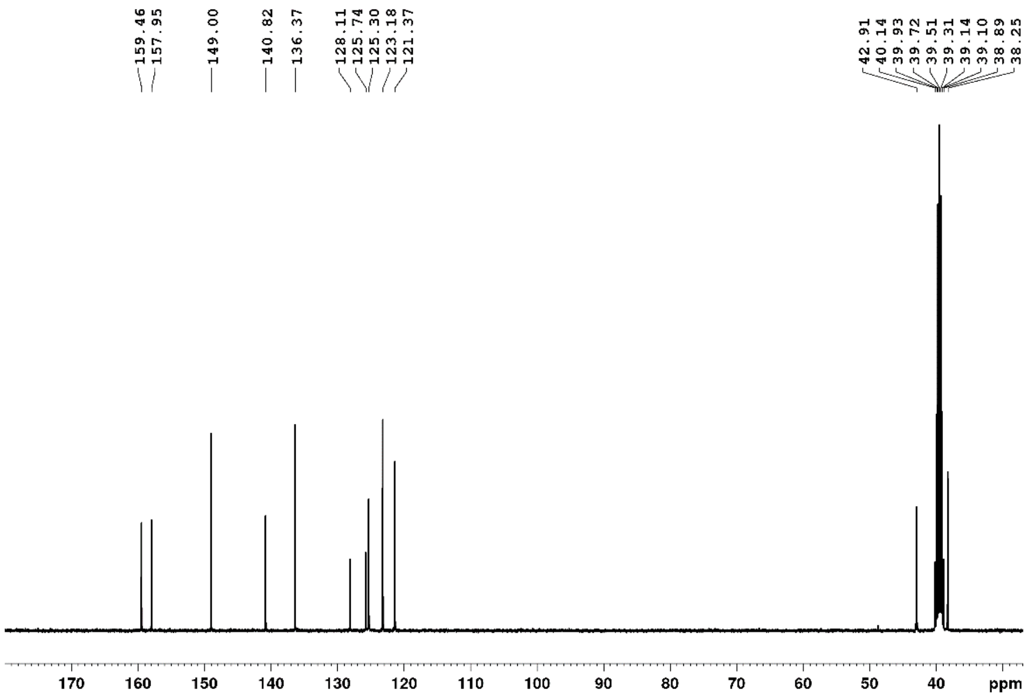

Figure S10. *^{13}C solution-state NMR spectrum (100 MHz) of **p2o1** solubilized in DMSO-d_6. Recorded on a Bruker Avance III HD 400 spectrometer. During acquisition, the number of scans was equal to 1024, while a repetition delay of 2 seconds was used.*

S4.3. ¹H-¹³C HSQC spectrum

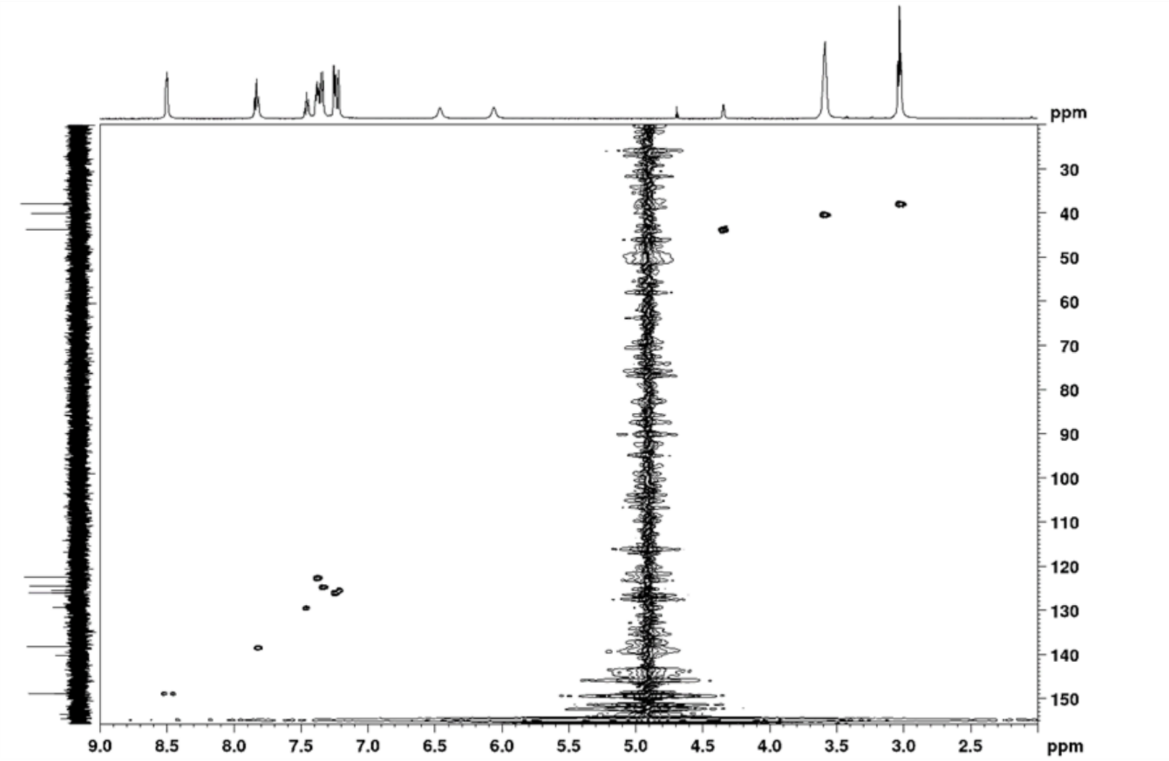

Figure S11. *^{1}H-^{13}C solution-state HSQC spectrum of the* ***p2o1*** *hydrogel in H_2O:D_2O (9:1) at a concentration of 1% w/V. During acquisition, the number of scans was equal to 2, while a repetition delay of 2 seconds was used.*

S4.4. ¹H-¹³C HMBC spectrum

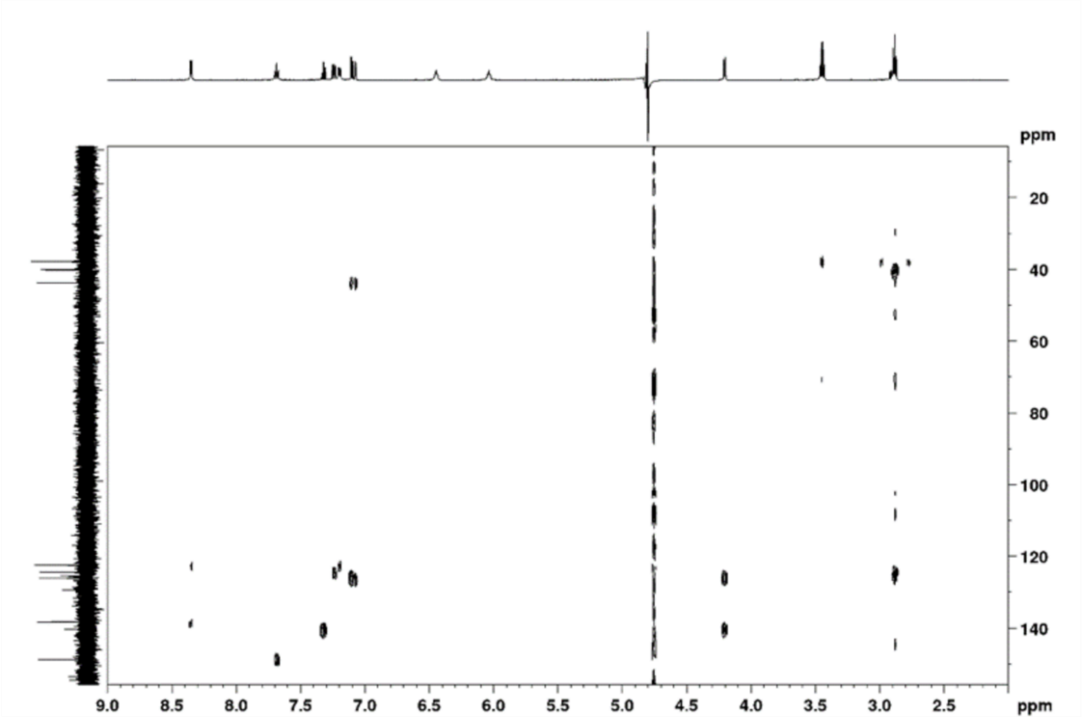

Figure S12. *^{1}H-^{13}C solution state HMBC spectrum of the* ***p2o1*** *hydrogel in H_2O:D_2O (9:1) at a concentration of 1% w/V. Water suppression was accomplished using the watergate pulse sequence. During acquisition, the number of scans was equal to 16, while a repetition delay of 1.5 seconds was used.*

S4.5. ^1H-^{15}N HSQC spectrum

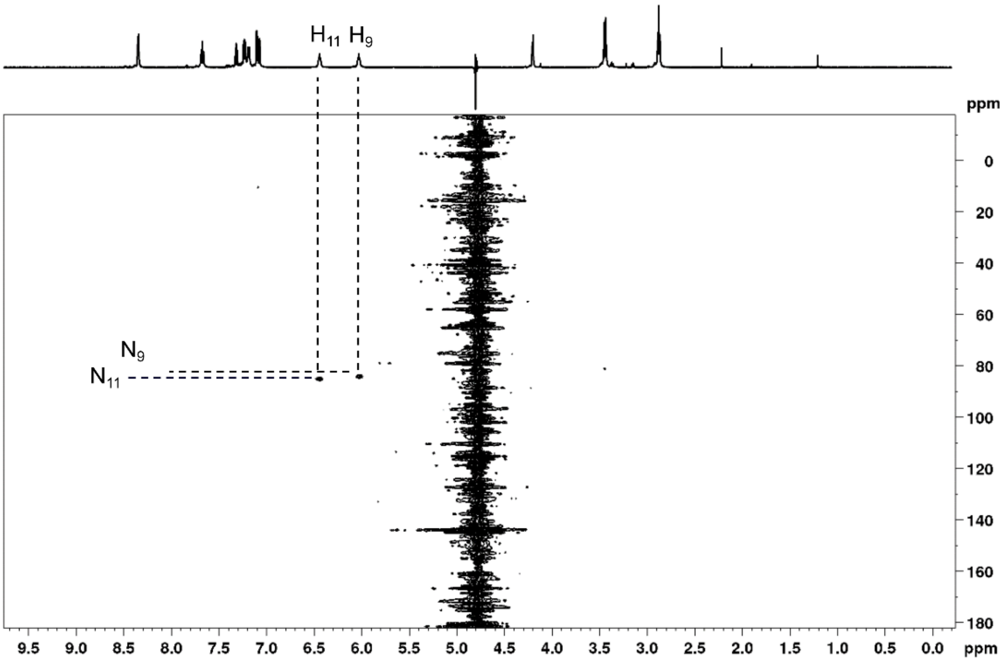

Figure S13. *^1H-^{15}N solution state HSQC spectrum of the* ***p2o1*** *hydrogel in H_2O:D_2O (9:1) at a concentration of 1% w/V. Water suppression was accomplished using the 3-9-19 pulse sequence. During acquisition, the number of scans was equal to 32, while a repetition delay of 3 seconds was used.*

S4.6. ^1H-^{13}C HSQC spectrum at 60 °C

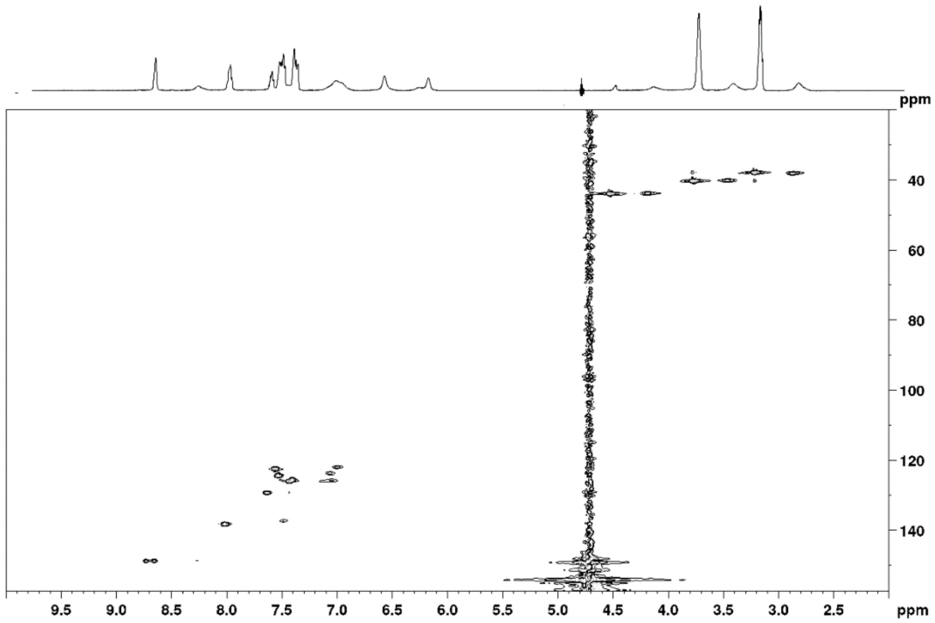

Figure S14. *^1H-^{13}C solution state HSQC spectrum of the* ***p2o1*** *hydrogel in H_2O:D_2O (9:1) at a concentration of 1% w/V and a temperature of 60 °C. Water suppression on the 1D spectrum shown above the 2D spectrum was accomplished through excitation sculpting. A similar cross peak pattern is observed for the additional broad peaks compared to the narrow peaks, indicating both signals originate from the same chemical structure but a different molecular phase. During acquisition, the number of scans was equal to 4, while a repetition delay of 2 seconds was used.*

S4.7. VT-NMR experiments

When performing VT-NMR experiments, it is paramount to obtain an accurate temperature control. For this reason, two-calibration curves were made, one focusing on the lower temperature region (282 K – 330 K) and one describing the higher temperature region (300 K – 380 K). The calibration curves were obtained using the Bruker NMR standard reference samples, 99.8% methanol-d_4 and 80% glycol in DMSO-d_6, respectively. Temperatures were calculated by means of the chemical shift difference between the aliphatic and hydroxyl protons using equations 4 and 5. Here, Δ_1 represents the frequency difference between the methyl and the hydroxyl signal of methanol, while Δ_2 represents the frequency difference between the methylene and hydroxyl signal of glycol. Sample preparation followed the guidelines described in the General Details (Section 4.1). Furthermore, between each temperature, a 15 minute stabilization time was set to obtain a homogeneous temperature throughout the whole sample. In addition, between each measurement, shimming and tuning and matching of the probe was performed.

$$T_{282-330K} = -(16.7467 * \Delta_1 - 52.5130) * \Delta_1 + 419.1381 \quad (4)$$

$$T_{300-380K} = -99 * \Delta_2 + 463.00 \quad (5)$$

Besides a VT-NMR experiment to study the gel-to-sol transition, *i.e.* an experiment starting at low temperature and increasing the temperature until the sample has reached the solution phase, a VT-NMR experiment was performed to study the sol-to-gel transition. To achieve this, a 1.0% w/V mixture of the gelator **p2*o*1** in D_2O:H_2O (9:1) was heated in a closed vial at 120 °C to reach the solution state. Next, the hot solution was quickly transferred into an NMR tube and placed in the NMR magnet bore at a temperature of 80 °C. ^{1}H NMR spectra were recorded from 80 °C until 20 °C in steps of 10 °C (Figure S15). Furthermore, here, additional peaks appear in the spectrum between 70 °C and 40 °C, suggesting to gel-to-sol and sol-to-gel transition to occur through a similar pathway.

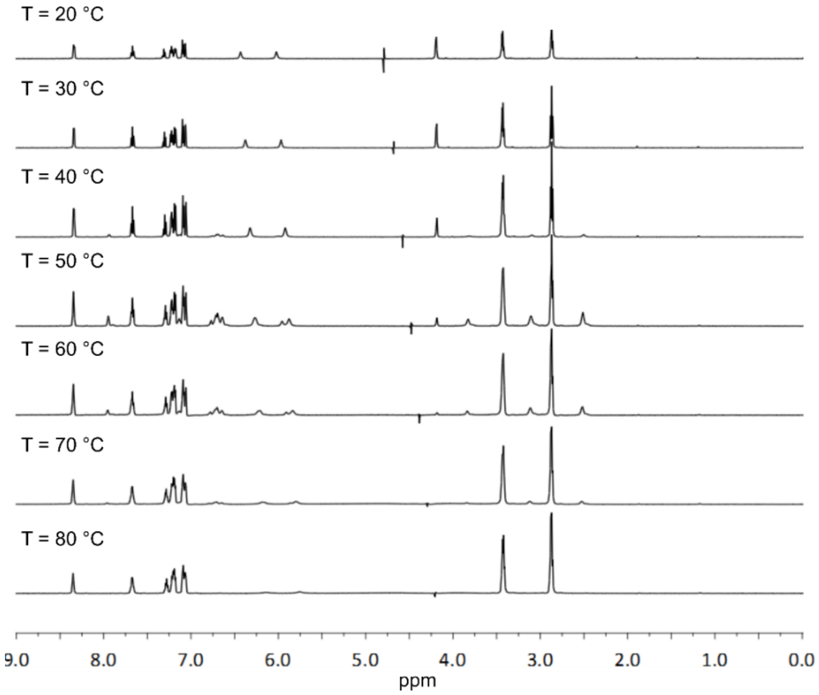

Figure S15. *^{1}H solution state VT-NMR experiment of a **p2o1** hydrogel sample in H_2O:D_2O (9:1) at a concentration of 1.0% w/V, starting at 80 °C and cooling down to 20 °C in steps of 10 °C. Water suppression was accomplished through excitation sculpting. During acquisition, the number of scans was equal to 16, while a repetition delay of 1 second was used for each experiment.*

S4.8. NOESY spectrum at 60 °C

From the VT-NMR experiments, it became clear that at 60 °C, an aggregated molecular phase of the gelator molecules exists. To study the possible noncovalent inter- and intramolecular noncovalent interactions present in these gelator aggregates, a NOESY spectrum was recorded of a hydrogel sample at 60 °C with a mixing time of 0.1 s.

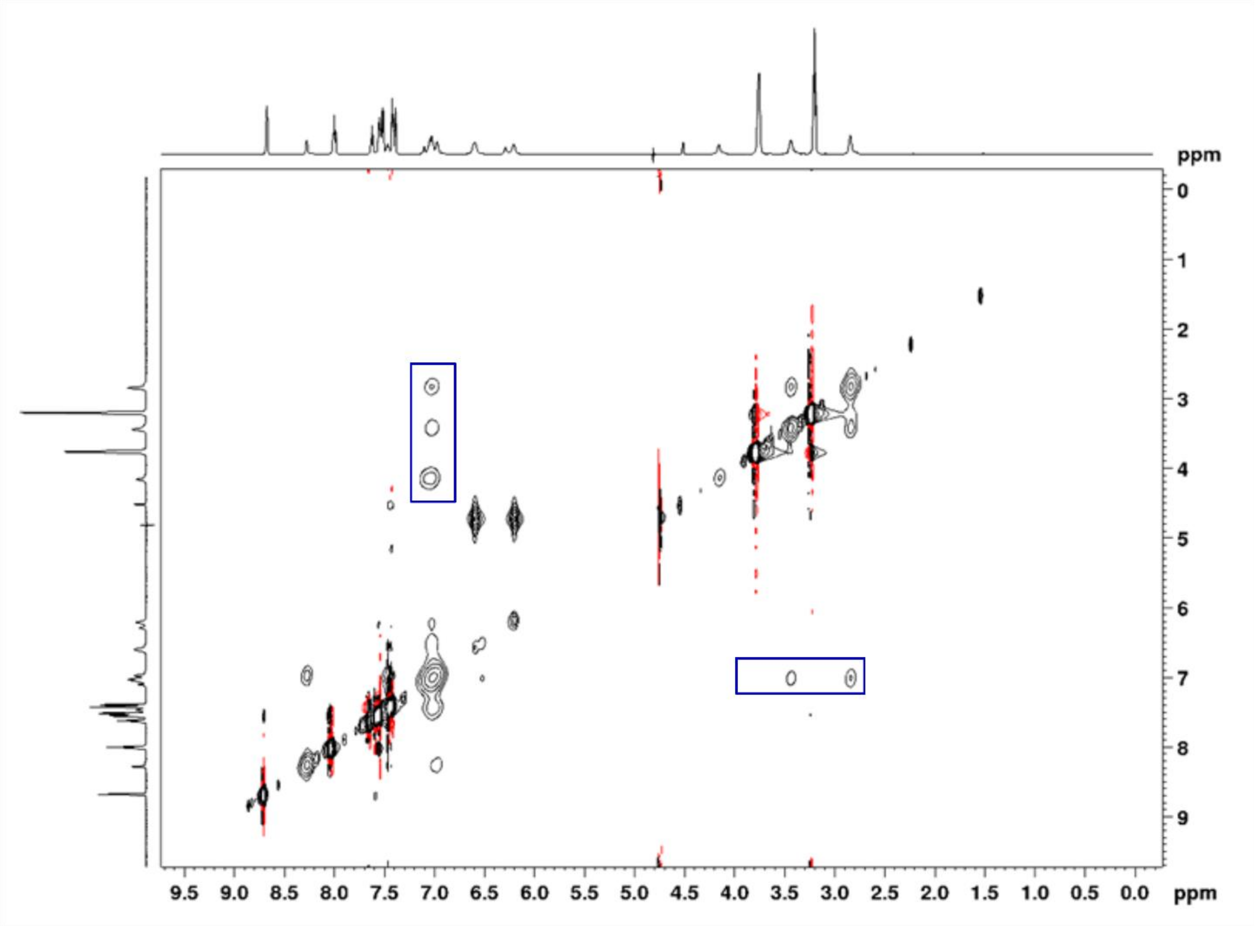

Figure S16. *^{1}H-^{1}H solution state NOESY of the **p2o1** hydrogel in H_2O:D_2O (9:1) at a concentration of 1.0% w/V at 60 °C, obtained with a mixing time of 0.1 s. The blue boxes highlight cross-peaks which indicate intermolecular interactions. All other cross-peaks can be explained by intramolecular proximity of the hydrogens. Water suppression was accomplished through excitation sculpting. Note that the cross-peaks in this spectrum have the same sign (negative) as the diagonal peaks. During acquisition, the number of scans was equal to 16, while a repetition delay of 1.5 seconds was used.*

S4.9. VC-NMR experiments

To validate that the broader set of signals originate from aggregates of the gelator molecule, a set of variable concentration NMR experiments was performed (Figure S17). From these results, we can conclude that at lower concentrations of gelators (0.5% w/V) the broad set of peaks does not appear at 60 °C. On the other hand, at higher concentrations of gelator (1.0 or 1.5% w/V) the broader set of peaks do appear. This further strengthens the hypothesis that the broader set of signals are caused by aggregate formation of the gelator **p2*o*1** in water.

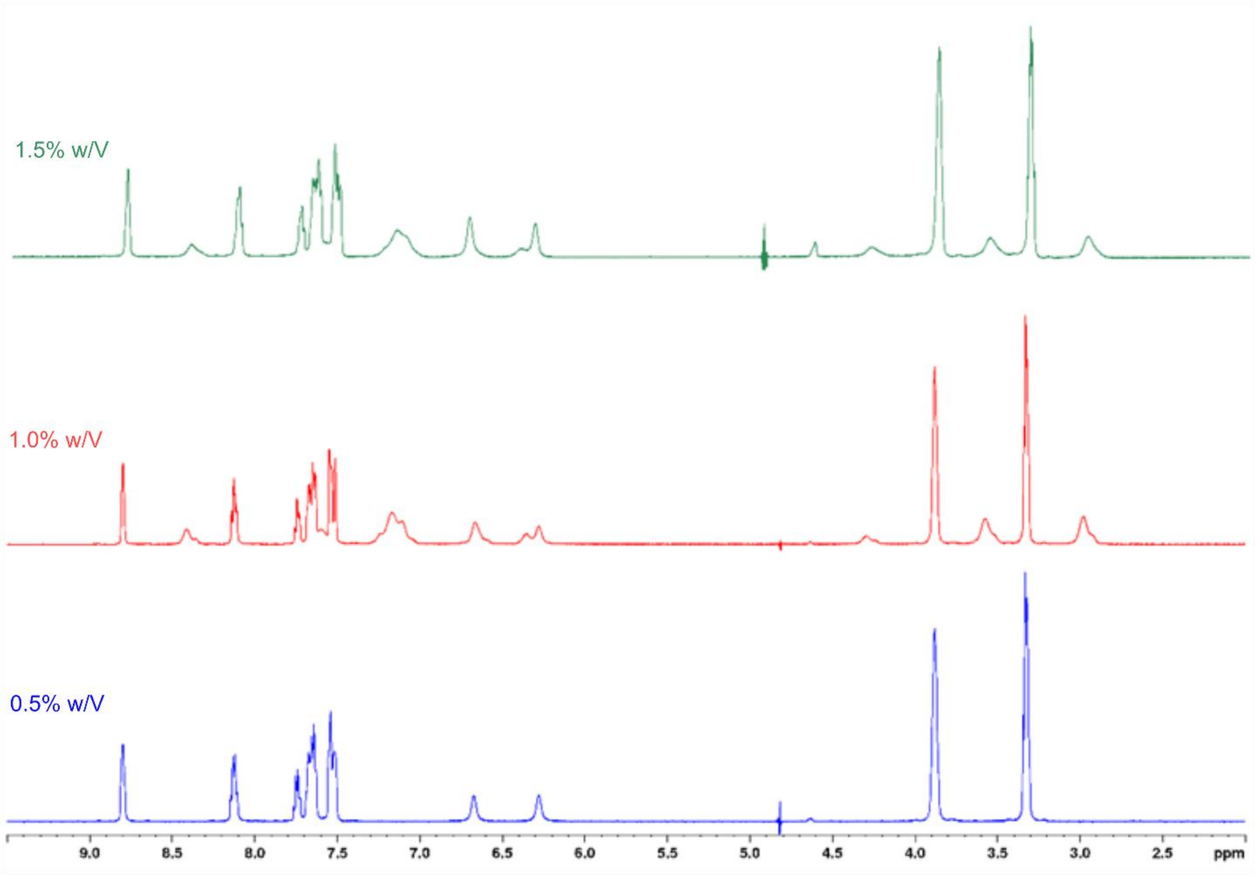

Figure S17. *^{1}H solution-state NMR spectrum of the* ***p2o1*** *hydrogel in H_2O:D_2O (9:1) measured at 60 °C with variable concentrations of gelator: 1.5% w/V = green, 1.0% w/V = red and 0.5% w/V = blue. During acquisition, the number of scans was equal to 16, while a repetition delay of 1.5 seconds was used for each experiment.*

S4.10. qNMR through ERETIC2

The quantification of a concentration in an unknown sample through the external qNMR method ERETIC2 is based on the following relationship:

$$(6) \qquad C_{unk} = kC_{ref}\frac{A_{unk}T_{unk}\theta_{unk}n_{ref}}{A_{ref}T_{ref}\theta_{ref}n_{unk}}$$

Here, the subscripts *unk* and *ref* label the properties associated with the unknown sample and reference sample respectively, with *C* the concentration of the solute, *A* the integral of a selected peak, *T* the temperature, ϑ the pulse length, *n* the number of scans and *k* a correction factor taking into account variances between measurements conducted on the reference sample and the unknown sample such as the differences in receiver gains [12]. In all ERETIC2 experiments performed in this study, *k* is equal to 1 as acquisition parameters remained constant and experiments were conducted without water suppression. Furthermore, concentration measurements were performed in triplicate by a 30° pulse. In total 5 transients were recorded with a relaxation delay (d1) of 30 seconds to assure > 99% recovery of the signal between every transient. The pulse length was corrected *via* the Bruker AU program PULSECAL before each measurement. All other acquisition and processing parameters remained constant for each experiment.

To assess the accuracy of the ERETIC2 method, two standardized samples were made in DMSO-d_6 being:

ethyl benzoate (6.99 ± 0.07 mM, flame sealed NMR tube) and isopropylacetate (6.70 ± 0.07 mM). For the **p2*o*1** gel samples, a 20 mL 1.0% w/V stock solution was made in D_2O which was heated until the gelator was fully solubilized, after which 0.5 mL of the stock solution was added to a 5 mm NMR tube.

A heterogeneous NMR sample, such as a gel, can lead to magnetic field inhomogeneity in the sample. This might cause incorrect integrations of resonance peaks, which is crucial for quantitative NMR experiments. To test the influence of an inhomogeneous magnetic field on the accuracy of the ERETIC2 method, the concentration of the standardized ethyl benzoate sample in DMSO-d_6 (6.99 ± 0.07 mM) was calculated in an artificially generated inhomogeneous field and compared to its adequately shimmed spectrum. Peaks at 7.97, 7.66, 7.53 and 4.33 ppm were integrated and used for quantification. The experiment was performed in triplicate and the sample was shimmed between each measurement. The concentration of the ethyl benzoate sample in DMSO-d_6 determined through ERETIC2 in presence of an artificial inhomogeneous magnetic field is equal to 7.17 ± 0.13 mM. Compared to the actual concentration, it can be stated that an inhomogeneous field results in a relatively small percentage error on the concentration of 1.93%. Hence, it is concluded that the inhomogeneity induced by the gel sample will insignificantly influence the accuracy of the ERETIC2 method.

Besides an inhomogeneous field, the temperature at which the measurements of the unknown and reference sample take place could also affect the accuracy of ERETIC2. Indeed, Wider and Dreier observed that measuring the concentration of a sample of interest at different temperatures with a reference spectrum at a single temperature, is only valid until 60 °C.[13] To evaluate this observation, the concentration of the ethyl benzoate sample was calculated with the ERETIC2 method at different temperatures (20, 40, 60, 80 °C) using the ethyl benzoate spectrum at 20 °C as a reference (method 1). For each temperature, the measurement was performed in triplicate and between experiments the sample was removed from the magnet and reinserted, the probe was tuned and matched, the sample was shimmed and a 90° pulse was calibrated. The results show that indeed accurate concentrations could be determined until 60 °C. However, at 80 °C the error on the concentration increased significantly towards a percentage error of 13.5 %. (Table S2, Figure S18). To accurately determine concentrations at 80 °C, a different method is proposed where for each temperature of the unknown sample, a reference spectrum is used at the same temperature (method 2). Comparing the results of method 1 and method 2, it is concluded that method 2 provides significantly more accurate results, with a percentage error of only 0.42 % at 80 °C. In order to validate if this method would be usable when the reference sample and unknown sample contained a different molecule, the concentration of the isopropylacetate standardized sample in DMSO-d_6 was measured at 80 °C (aliphatic peaks at 4.89, 1.96 and 1.20 ppm were used for integration) with the ethyl benzoate spectrum at 80 °C serving as the reference. Using this method, an acceptable accuracy is achieved as an average concentration of 6.37 ± 0.08 mM (4.71 % error) is obtained.

Table S2. *Concentration (C) in mM and percentage error (Δ%) of the ethyl benzoate standardized sample in DMSO-d_6 calculated by means of ERETIC2 via method 1 or method 2. All experiments were performed in triplicate and hence standard deviations of the obtained properties is provided between brackets.*

	**T (°C)**	**20**	**40**	**60**	**80**
**Method 1**	**C (mM)**	6.95 (0.10)	6.80 (0.10)	6.76 (0.08)	6.05 (0.38)
	**Δ%**	0.554% (0.015)	2.730% (0.014)	3.243% (0.012)	13.51% (0.05)
**Method 2**	**C (mM)**	6.99 (0.105)	7.09 (0.14)	7.10 (0.0732)	7.02 (0.0559)
	**Δ%**	0.021% (0.014)	1.460% (0.014)	1.524% (0.0120)	0.42% (0.05)

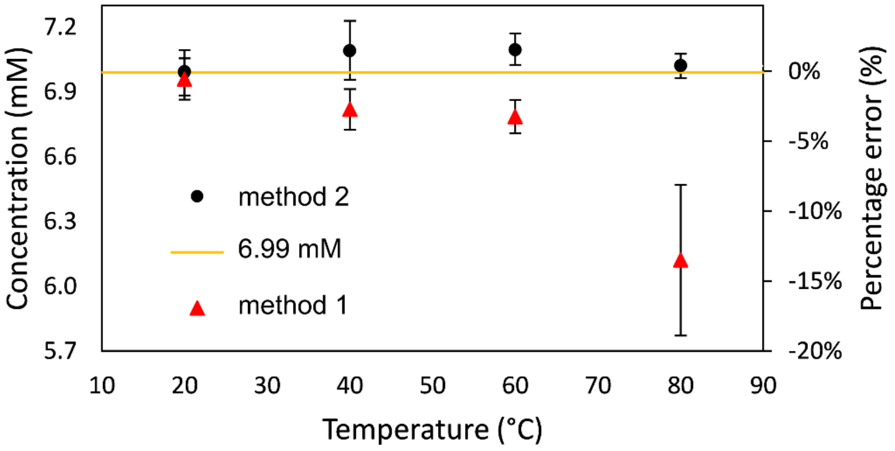

Figure S18. *Concentration and percentage error for standardized ethyl benzoate sample (6.99 ± 0.07 mM), determined through ERETIC2 via method 1 (red triangle) or method 2 (black dot) at different temperatures.*

Having established ERETIC2 as an accurate technique to quantify the unknown concentrations of a specific molecule in a heterogeneous sample at different temperatures, we set out to use it to track the hydrogelation mechanism of **p2*o*1**. Concentrations of the different gelator phases, *i.e.* narrow peaks (solvated gelators) and broad peaks (aggregated gelators), were calculated at temperatures ranging from 20 °C to 80 °C with a step of 20 °C. In all cases, the reference sample (ethyl benzoate in DMSO-d_6) was measured at the same temperature (method 2). Experiments were performed in triplicate to assess the deviations.

Table S3. *Concentration (C) in mM and percentage of gelator* ***p2o1*** *visible via solution-state ^{1}H NMR. A differentiation is made between the broader set of signals (indicating the gelator in an aggregated state) and the narrow set of signals (indicating the gelators to be in the solvated state). Three separate gel samples were measured to obtain an average result with standard deviation (provided between brackets). For each separate gel sample, a standard deviation on the measured properties is provided as all non-overlapping ^{1}H signals of the gelator spectrum (H-2, H-12, H-8 and H-7) were used for integration. These standard deviations are also provided between brackets. Additionally, the sum of the broad and narrow set of peaks is provided. The total concentration of gelator in each sample, including all possible material states (solvated, aggregated, immobilized) is equal to 23.1 mM, which was used to calculate the percentages.*

	**T (°C)**	**20**	**40**	**60**	**80**
**Gel 1**	**C (mM)**	0 (0)	0 (0)	2.05 (0.13)	8.47 (0.0771)
broad	**%**	0% (0)	0 (0)	8.89% (0.005)	36.6% (0.00333)
**Gel 2**	**C (mM)**	0 (0)	0% (0)	2.10 (0.11)	9.73 (0.322)
broad	**%**	0 (0)	0 (0)	9.08% (0.005)	42.1% (0.0139)
**Gel 3**	**C (mM)**	0 (0)	0 (0)	2.20 (0.2)	4.66 (0.449)
broad	**%**	0% (0)	0% (0)	9.52% (0.013)	20.16% (0.0114)
**Gel 1**	**C (mM)**	2.13 (0.65)	2.5 (0.8)	8.38 (0.3)	9.89 (0.904)
narrow	**%**	9.2% (0.2)	10.81% (0.04)	36.2% (0.012)	42.8% (0.0391)
**Gel 2**	**C (mM)**	1.8 (0.3)	3.75 (0.598	8.62 (0.27)	11.4 (0.223)
narrow	**%**	8.191% (0.012)	16.2% (0.0259)	37.3% (0.012)	49.4% (0.00963)
**Gel 3**	**C (mM)**	0 (0)	0 (0)	7.10 (0.07)	7.02 (0.0559)
narrow	**%**	0% (0)	0% (0)	-1.52% (0.012)	-0.418% (0.0539)
**Gel 1**	**C (mM)**	2.1 (0.7)	2.49 (0.817)	10.4 (0.3)	18.4 (0.875)
sum	**%**	9.20% (0.02)	10.8 % (0.0353)	45.1% (0.014)	79.4% (0.0143
**Gel 2**	**C (mM)**	1.9 (0.3)	3.75 (0.598)	10.7 (0.3)	21.2 (0.543)
sum	**%**	8.192% (0.013)	16.24% (0.03)	46.33% (0.013)	91.48% (0.0235)

**Gel 3**	**C (mM)**	1.9 (0.3)	3.6 (1.3)	10.7 (0.5)	16.1 (0.710)
sum	**%**	8.161% (0.014)	15.71% (0.06)	46.2% (0.02)	69.8% (0.0307)
**Average**	**C (mM)**	0 (0)	0 (0)	2.12 (0.168)	7.62 (2.21)
broad	**%**	0% (0)	0% (0)	9.16% (0.00724)	33.0% (0.0954)
**Average**	**C (mM)**	2.0 (0.5)	3.33 (1.09)	8.49 (0.294)	10.9 (0.922)
narrow	**%**	8.51% (0.02)	14.4% (0.0473)	36.7% (0.0127)	47.3% (0.0399)
**Average**	**C (mM)**	2.0 (0.5)	3.33 (1.09)	10.6 (0.396)	18.6 (2.20)
sum	**%**	8.51% (0.0197)	14.4% (0.0473)	45.9% (0.0171)	80.2% (0.0951)

S4.11. NMR experiments below MGC

As some results indicated the narrow ^{1}H signals of the **p2*o*1** hydrogelator to contain information on the gel phase, a number of comparative NMR experiments were performed between a fully gelled **p2*o*1** sample and a diluted **p2*o*1** sample below the minimum gelation concentration (MGC) and in absence of interfering solid particles. To obtain the latter sample, 5 mg of **p2*o*1** was mixed with 0.5 mL pure D_2O. Next, the mixture was pushed through a Micropur 25 mm PTFE syringe filter with a 0.45 µm pore size filter to remove all solid particles larger than 0.45 µm. The resulting solution appeared transparent. To ensure this sample was below the MGC, we tested whether it was possible for the sample to pass the vial inversion test after a heat-and-cool cycle. This was not the case, indicating that the **p2*o*1** concentration in this sample was far below the MGC. From previous results, it is known that for a heat-and-cool cycle gelation procedure in water, the MGC of **p2*o*1** is equal to 0.4% w/V [7].

Table S4. *Transverse relaxation constants (T_2) expressed in seconds for the narrow* ***p2o1*** *aliphatic ^{1}H signals at 4.11, 3.35 and 2.79 ppm for a fully gelled sample and a diluted sample below the MGC at room temperature (22.5 °C). A Carr-Purcell-Meiboom-Gill (CPMG) experiment was performed at room temperature in triplicate to obtain these values together with a standard deviation (provided between brackets). Pure D_2O was used for both samples to omit the need for solvent suppression during the experiment.*

**ppm**	**4.11** (12-H)	**3.35** (7-H)	**2.79** (8-H)
***T_2*, gel sample (s)**	0.324 (0.018)	0.20 (0.06)	0.122 (0.007
***T_2*, diluted sample (s)**	0.441 (0.009)	0.36 (0.07)	0.37 (0.05)

For all three ^{1}H-signals, a lower T_2 value is observed for the gel sample compared to the diluted sample. This indicates that the narrow ^{1}H-peaks observable in the gel sample are characterized by an increased rotational correlation time. In other words, these resonance signals contain information of a larger molecular structure compared to the corresponding signals in the diluted sample.

S5. References and notes

1. Louis, B.; Camacho, R.; Bresolí-Obach, R.; Abakumov, S.; Vandaele, J.; Kudo, T.; Masuhara, H.; Scheblykin, I.G.; Hofkens, J.; Rocha, S. Fast-tracking of single emitters in large volumes with nanometer precision. *Opt. Express* **2020**, *28*, 28656-28671, doi:10.1364/OE.401557.
2. Abraham, M.J.; Murtola, T.; Schulz, R.; Páll, S.; Smith, J.C.; Hess, B.; Lindahl, E. GROMACS: High performance molecular simulations through multi-level parallelism from laptops to supercomputers. *SoftwareX* **2015**, *1-2*, 19-25, doi:https://doi.org/10.1016/j.softx.2015.06.001.
3. Martinez, L.; Andrade, R.; Birgin, E.G.; Martinez, J.M. PACKMOL: A Package for Building Initial Configurations for Molecular Dynamics Simulations. *J. Comput. Chem.* **2009**, *30*, 2157-2164, doi:10.1002/jcc.21224.
4. MacKerell, A.D.; Bashford, D.; Bellott, M.; Dunbrack, R.L.; Evanseck, J.D.; Field, M.J.; Fischer, S.; Gao, J.; Guo, H.; Ha, S.; et al. All-Atom Empirical Potential for Molecular Modeling and Dynamics Studies of Proteins. *J. Phys. Chem. B* **1998**, *102*, 3586-3616, doi:10.1021/jp973084f.

5. Foloppe, N.; MacKerell, J.A.D. All-atom empirical force field for nucleic acids: I. Parameter optimization based on small molecule and condensed phase macromolecular target data. *J. Comput. Chem.* **2000**, *21*, 86-104, doi:10.1002/(SICI)1096-987X(20000130)21:2<86::AID-JCC2>3.0.CO;2-G.
6. Zoete, V.; Cuendet, M.A.; Grosdidier, A.; Michielin, O. SwissParam: a fast force field generation tool for small organic molecules. *J. Comput. Chem.* **2011**, *32*, 2359-2368, doi:10.1002/jcc.21816.
7. Van Lommel, R.; Rutgeerts, L.A.J.; De Borggraeve, W.M.; De Proft, F.; Alonso, M. Rationalising Supramolecular Hydrogelation of Bis-Urea-Based Gelators through a Multiscale Approach. *ChemPlusChem* **2020**, *85*, 267-276, doi:10.1002/cplu.201900551.
8. Berendsen, H.J.C.; Postma, J.P.M.; van Gunsteren, W.F.; DiNola, A.; Haak, J.R. Molecular dynamics with coupling to an external bath. *J. Chem. Phys.* **1984**, *81*, 3684-3690, doi:10.1063/1.448118.
9. Parrinello, M.; Rahman, A. Polymorphic transitions in single crystals: A new molecular dynamics method. *J. Appl. Phys.* **1981**, *52*, 7182-7190, doi:10.1063/1.328693.
10. Van Lommel, R.; Zhao, J.; De Borggraeve, W.M.; De Proft, F.; Alonso, M. Molecular dynamics based descriptors for predicting supramolecular gelation. *Chem. Sci.* **2020**, *11*, 4226-4238, doi:10.1039/D0SC00129E.
11. Hwang, T.L.; Shaka, A.J. Water Suppression That Works. Excitation Sculpting Using Arbitrary Wave-Forms and Pulsed-Field Gradients. *J. Magn. Reson., Series A* **1995**, *112*, 275-279, doi:https://doi.org/10.1006/jmra.1995.1047.
12. Lagerquist, L.; Rahkila, J.; Eklund, P. Utilization of 31P PULCON for Quantitative Hydroxyl Group Determination in Lignin by NMR Spectroscopy. *ACS Sust. Chem. Eng.* **2019**, *7*, 9002-9006, doi:10.1021/acssuschemeng.9b01269.
13. Wider, G.; Dreier, L. Measuring Protein Concentrations by NMR Spectroscopy. *J. Am. Chem. Soc.* **2006**, *128*, 2571-2576, doi:10.1021/ja055336t.
